# Generative artificial intelligence: Can ChatGPT write a quality abstract?

**DOI:** 10.1111/1742-6723.14233

**Published:** 2023-05-04

**Authors:** Franz E Babl, Maximilian P Babl

**Affiliations:** ^1^ Emergency Department The Royal Children's Hospital Melbourne Victoria Australia; ^2^ Clinical Sciences Murdoch Children's Research Institute Melbourne Victoria Australia; ^3^ Department of Paediatrics, Faculty of Medicine, Dentistry and Health Sciences The University of Melbourne Melbourne Victoria Australia; ^4^ Department of Critical Care, Faculty of Medicine, Dentistry and Health Sciences The University of Melbourne Melbourne Victoria Australia; ^5^ Xavier College Melbourne Victoria Australia

**Keywords:** artificial intelligence, ChatGPT, generative artificial intelligence

## Abstract

ChatGPT is a generative artificial intelligence chatbot which may have a role in medicine and science. We investigated if the freely available version of ChatGPT can produce a quality conference abstract using a fictitious but accurately calculated data table as applied by a non‐medically trained person. The resulting abstract was well written without obvious errors and followed the abstract instructions. One of the references was fictitious, known as ‘hallucination’. ChatGPT or similar programmes, with careful review of the product by authors, may become a valuable scientific writing tool. The scientific and medical use of generative artificial intelligence, however, raises many questions.

**Key findings**

- We investigated if ChatGPT can create a quality conference abstract using a fictitious data set.
- The resulting abstract was well written without obvious errors. One of the references was made up.
- Generative artificial intelligence like ChatGPT may become a valuable writing tool but also raises many questions.

Generative artificial intelligence (AI) is capturing global attention and multiple uses in science and medicine have been suggested.^1^, ^2^, ^3^, ^4^ A readily available system is the recently launched ChatGPT (Chat Generative Pre‐trained Transformer). It produces human‐like text trained on data from the internet originally written by humans, created by OpenAI, San Francisco. ChatGPT uses a typed or pasted ‘prompt’ which the chatbot responds to within seconds using natural language. This exchange can be continued and refined with additional ‘prompts’.^2^ Since its release in November 2022, ChatGPT has been used by more than 100 million people. ChatGPT‐3 is free and can be optimised for speed with a subscription; a new more powerful version GPT‐4 was released in March 2023.^2^

We investigated if the freely available version of ChatGPT can produce a grammatically and structurally correct conference abstract of reasonable quality, based on a standard set of instructions with a data set provided (Box 1). To avoid ChatGPT extracting from previously published abstracts or data, we used an adult disease with no data in children and created a fictitious, but accurately calculated data table. ChatGPT was applied by a non‐medically trained person (MPB) familiar with ChatGPT, based on the initial prompt and was not further corrected.

BOX 1 **Abstract prompt and ChatGPT response**

**Prompt**

Write a 250 word abstract on plantar fasciitis in children with title, introduction, methods, results and discussion section using the data below. Add 2 references.

Double blind RCT comparing oral prednisolone and placebo for 10 days. Intention to treat. 89 in placebo group, 88 in steroid group. Children aged 8–12 years old. Outcome: return of normal foot function at 1, 3 and 6 months. No adverse events.

**Table:** 

Group	Time (months)	*n*	*n*	%	(95%CI)
Placebo	1	40	89	44.94	(34.38–55.86)
Placebo	3	45	85	52.94	(41.81–63.87)
Placebo	6	50	82	60.98	(49.57–71.56)
Prednisolone	1	60	90	66.67	(55.95–76.26)
Prednisolone	3	72	88	81.82	(72.16–89.24)
Prednisolone	6	84	85	98.82	(93.62–99.97)

Comparison

1 month OR = 2.45 (1.34–4.49), *P* = 0.003.

3 months OR = 4.00 (2.01–7.97), *P* < 0.001.

6 months OR = 53.76 (7.13–405.63), *P* < 0.001.

**Response**

**Title: Efficacy of oral prednisolone for plantar fasciitis in children: A double‐blind randomized controlled trial**

**Introduction**: Plantar fasciitis is a common cause of heel pain in adults, but it is rarely reported in children. However, recent studies have shown that plantar fasciitis is a common cause of heel pain in children aged 8–12 years old. Although there is no standard treatment for plantar fasciitis in children, non‐steroidal anti‐inflammatory drugs and rest are commonly used. The aim of this study is to investigate the efficacy of oral prednisolone in treating plantar fasciitis in children.

**Methods**: A double‐blind randomized controlled trial was conducted on 177 children aged 8–12 years old, who were randomly assigned to receive either oral prednisolone or placebo for 10 days. The outcome measure was the return of normal foot function at 1, 3 and 6 months after treatment. The study was conducted with intention to treat, and no adverse events were reported.

**Results**: At 1, 3 and 6 months after treatment, the percentage of children who had returned to normal foot function was significantly higher in the prednisolone group than in the placebo group (66.67% *vs* 44.94%, 81.82% *vs* 52.94% and 98.82% *vs* 60.98%, respectively). The odds ratios for the return of normal foot function at 1, 3 and 6 months were 2.45 (95% CI 1.34–4.49, *P* = 0.003), 4.00 (95% CI 2.01–7.97, *P* < 0.001) and 53.76 (95% CI 7.13–405.63, *P* < 0.001), respectively.

**Discussion**: Our study suggests that oral prednisolone may be an effective treatment for plantar fasciitis in children. However, further studies are needed to confirm these findings and to investigate the long‐term effects of oral prednisolone on foot function in children with plantar fasciitis.

**References**:

Yan S, Li B, Hu Y *et al*. A systematic review and meta‐analysis of risk factors for pediatric plantar fasciitis. *J. Foot Ankle Res*. 2020; **13**: 16. doi: 10.1186/s13047‐020‐0397‐1

Radford JA, Landorf KB, Buchbinder R, Cook C. Effectiveness of low‐dye taping for the short‐term treatment of plantar heel pain: a randomised trial. *BMC Musculoskelet. Disord*. 2006; **7**: 64. doi: 10.1186/1471‐2474‐7‐64

The resulting abstract is impressive: it is well written with headings and word length as requested. The title is appropriate, the introductory sentences are consistent with the state of knowledge on plantar fasciitis, the results including the statistical tests were correctly extracted from the data table, and the conclusions are a reasonable interpretation of the results. There are no obvious errors. The references seem appropriate but only the second reference is real with the first reference fictitious; this has been noted before for ChatGPT and has been termed ‘hallucinations’.^2^

The impact of generative AI in medicine is potentially revolutionary with proposed areas of use shown in Box 2.^1^, ^2^, ^4^ As this small experiment has shown, ChatGPT allows a non‐scientist to produce a satisfactory conference abstract. We doubt even the current, uncorrected version would be rejected by an abstract assessment committee. Editorials and letters using ChatGPT have already been published.^5^, ^6^

BOX 2 **Some current and possible future uses of artificial intelligence in medicine^1^, ^2^, ^3^, ^4^**

**Clinical medicine**

Analysis of medical images

Detection of drug interactions

Identifying high‐risk patients

Coding of medical notes

Medical note taking

Medical consultation

Retrieval of medical information

Electronic medical record decision support

Translation of foreign languages

Explanation of laboratory and imaging tests

**Research**

Improved trial performance, for example via decision support, improved recruitment of participants, outcome monitoring

Analysis and interpretation of large research databases

Writing, revision and summarising of scientific papers and grants

**Other uses**

Role in public health, for example identify outbreaks

Assessor and teacher in medical education

Hospital business operations

ChatGPT or similar programmes when used as a writing tool with careful review by scientific authors may become a valuable tools to speed a first draft of papers and grants, summarise or simplify long articles, reduce duplication, polish and streamline existing documents or assist researchers with limited English skills (although it also works in other languages). It does not seem obvious why standard methodology or even results sections of a paper could not be written with the assistance of generative AI when large sections of research papers are proscribed in some detail – often with suggested wording by various EQUATOR reporting guidelines.^7^ EQUATOR now includes guidance for studies including AI and machine learning.

Advantages of AI in medicine will need to be balanced with possible legal, ethical and patient safety challenges.^1^, ^3^, ^4^ In its scientific use, there are many unanswered questions and issues: what are the ethical limits of its use and does its use need to be disclosed? Can or should AI like ChatGPT be listed as an author and how can its use be acknowledged? Recently published guidance by the World Association of Medical Editors states that chatbots cannot be authors and that authors be transparent when they are used.^8^ AI may plagiarise the work of others; even designated tools to detect AI may be limited in their ability to detect AI written text and may falsely attribute human written text to AI.^4^ Who is responsible for mistakes or harmful advice created by AI? Are data entered into ChatGPT and its outputs accessible to others as it is drawing on existing information and learning from its users? Could it lead to the amplification of biases, such as racial, sex and other stereotypes, or fuel the spread of misinformation?^3^, ^6^, ^8^

It seems likely that AI, which is constantly evolving and improving, will be widely incorporated into medicine in general and scientific writing in particular. Researchers, journals and funding agencies will need to rapidly come to terms with it.

## Data Availability

No data are available.

## References

[1] Haug CJ , Drazen JM . Artificial intelligence and machine learning in clinical medicine, 2023. N. Engl. J. Med. 2023; 388: 1201–8. 36988595 10.1056/NEJMra2302038

[2] Lee P , Bubeck S , Petro J . Benefits, limits, and risks of GPT‐4 as an AI chatbot for medicine. N. Engl. J. Med. 2023; 388: 1233–9. 36988602 10.1056/NEJMsr2214184

[3] The Lancet Digital Health . ChatGPT: friend or foe? Lancet Digit. Health 2023; 5: e102. 36754723 10.1016/S2589-7500(23)00023-7

[4] Stokel‐Walker C , Van Noorden R . The promise and peril of generative AI. Nature 2023; 614: 214–6. 36747115 10.1038/d41586-023-00340-6

[5] Curtis N , ChatGPT . To ChatGPT or not to ChatGPT? The impact of artificial intelligence on academic publishing. Pediatr. Infect. Dis. J. 2023; 42: 275. 36757192 10.1097/INF.0000000000003852

[6] D'Amico RS , White TG , Shah HA , Langer DJ . I asked a ChatGPT to write an editorial about how we can incorporate chatbots into neurosurgical research and patient care…. Neurosurgery 2023; 92: 663–4. 36757199 10.1227/neu.0000000000002414

[7] The EQUATOR (Enhancing the QUAlity and Transparency Of health Research) . [Cited 8 Apr 2023.] Available from URL: www.equator-network.org/

[8] Zielinski C , Winker M , Aggarwal R *et al*. *Chatbots, ChatGPT, and Scholarly Manuscripts*. WAME Recommendations on ChatGPT and Chatbots in Relation to Scholarly Publications. World Association of Medical Editors, 2023. [Cited 22 Feb 2023.] Available from URL: https://wame.org/page3.php?id=106 10.25259/NMJI_365_23 37615142

